# Supplementary material for: Participants’ Perspective of Engaging in a Gym-Based Health Service Delivered Secondary Stroke Prevention Program after TIA or Mild Stroke
Source: Int J Environ Res Public Health. 2021 Oct 30;18(21):11448. doi: 10.3390/ijerph182111448 (PMC8583419; doi:10.3390/ijerph182111448)
Supplement: Supplementary file 1 [file ijerph-18-11448-s001.zip › ijerph-1416716-supplementary/suppl/Supplementary material 1. Discussion guide for semi-structured interviews.pdf]

SUPPLEMENTARY MATERIAL 1

Participants' perspective of engaging in a gym-based health service delivered secondary stroke prevention program after TIA or mild stroke.

Interview guide

1. **Can you tell me about what exercise or being physically active meant to you before you had your TIA**?
    a. What were your thoughts on whether it was a good or important thing to do?
        i. What do you think has influenced these ideas?
    b. What feelings did you associate with exercise or being physically active?

2. **Can you tell me about the exercise or physical activity (including any paid or unpaid physical labour) you have done throughout your life?**
    a. What did you do as a child
    b. What did you do as a younger adult
    c. What were you doing just prior to your TIA

    (for a–c:)
    - What activities?
    -How often?
    - Where?
    - With whom?
    - How did you feel about it?

    d. What has helped you to exercise in the past?
    e. What has prevented you from exercising or made it harder for you to exercise?

3. **What are your thoughts or feelings about exercising after your TIA?**
    a. What are your thoughts on whether it was a good or important thing to do?
        i. What do you think will happen if you exercise?
        ii. What do you think will happen if you don't exercise?
        iii. What impact do you think it will have on your risk of having another stroke?
    b. What are your thoughts on the type of exercise or how much exercise you should do?
    c. What has influenced these ideas?
    d. Can you tell me about any conversations you have had with your doctor or health professional about this?
        i. Who did you talk about this with?
        ii. What advice were you given about exercise or physical activity?

1. Advice re whether to exercise and why?
2. Advice re what to do?
3. How much to do?
4. How to do it?

iii. Can you tell me about any other sources of information you were provided or directed towards?

1. Websites
2. Community based resources
3. Information sheets

e. What additional advice or assistance do you would have been helpful?

4. Can you tell me about the exercise or physical activity you have been doing since your TIA?

a. What activities have you been doing?

i. For each identified ask:

1. How often?
2. Where do you do this?
3. Who do you do it with?
4. How do you feel when you are doing it?

5. Can you tell me what prevents you from exercising or makes it harder for you to exercise?

a. Any aspects of your physical health or abilities?
b. Mental health or the way you feel?
c. Knowledge or skills?
d. Access – things like transport or costs or access to the activity you would like to do
e. Attitudes or support of others?

6. Can you tell me what helps or would help you to exercise?

a. Any aspects of your physical health or abilities?
b. Mental health or the way you feel?
c. Knowledge or skills?

d. Access – things like transport or costs or access to the activity you would like to do

e. Attitudes or support of others?
