# Supplementary material for: Participants’ Perspective of Engaging in a Gym-Based Health Service Delivered Secondary Stroke Prevention Program after TIA or Mild Stroke
Source: Int J Environ Res Public Health. 2021 Oct 30;18(21):11448. doi: 10.3390/ijerph182111448 (PMC8583419; doi:10.3390/ijerph182111448)
Supplement: Supplementary file 1 [file ijerph-18-11448-s001.zip › ijerph-1416716-supplementary/suppl/SUPPLEMENTARY MATERIAL TABLE 1 example of coding tree.pdf]

SUPPLEMENTARY MATERIAL 1

Participants' perspective of engaging in a gym-based health service delivered secondary stroke prevention program after TIA or mild stroke.

TABLE 1. EXAMPLE OF CODING TREE

Initial narrative data to be coded	Initial open coding First level code (node)	Focused coding	Dimension	Subcategory	Category	Concept
They spent plenty of time with us to make sure we could do the exercise and that we were ok. They were constantly coming over check to see how your heart rate and breathing was going. You were always monitored so I didn't have any problems	Input from health professional<br><br>Feelings of safety<br><br>What I can do<br><br>Aspect of program	One on one support<br><br>Safety	Monitoring me	In person	Health professional support	What it offered me
A big part was for me was being around people that have been through that experience. It was great to have a shared experience. Knowing I'm not alone -it's probably therapy in itself	Part of the group<br><br>Sharing experiences<br><br>Aspects of program<br><br>Alone	Being with others<br><br>Companionship	Sharing experiences	Mutual support	Being in a group	What it offered me
I've got dreams and goals so I know what I want to do and where I want to go so now I want to get there and so being unhealthy is not going to help me do it	Determination<br><br>Value<br><br>Motivation to carry on	Doing it for the family	Connecting with my why	Thinking differently	Making changes	What I got out of it
It let me see that I could do things that I didn't think I could do. I didn't think I'd be ever able to do the things in the gym, which was probably a ridiculous notion on my part but	Reasoning<br><br>Motivation to continue<br><br>Understanding	Taking charge	Gaining confidence	Psychological factors	Feeling better	What I got out of it

that was how I felt						
